# Evaluation of the Antioxidant Activities and Tyrosinase Inhibitory Property from Mycelium Culture Extracts

**DOI:** 10.1155/2015/616298

**Published:** 2015-08-05

**Authors:** Ki Moon Park, Kyung Min Kwon, Seung Ho Lee

**Affiliations:** ^1^Department of Food Science and Biotechnology, Sungkyunkwan University, Suwon 440-746, Republic of Korea; ^2^Major of Nano-Bioengineering, Incheon National University, Incheon 406-772, Republic of Korea

## Abstract

Since mushrooms have many bioactive components, they have been used as components in folk medicine. Because mycelium has an advantage when it comes to large-scale production, this study aimed to evaluate the antioxidant properties and anti-tyrosinase activity from 55 mycelia in culture media. Relatively high 2,2-diphenyl-1-picrylhydrazyl (DPPH) scavenging capacity was detected from the ethanol extract of culture media including mycelium (EECiM) of *Morchella esculenta* var. *esculenta* (MEVE), *Auricularia polytricha* (APO), *Tremella aurantia* (TAU), *Volvariella bombycina* (VBO), and *Oudemansiella* sp. (Osp), which also showed strong reducing power and inhibitory activity in relation to the thiobarbituric acid (TBA) value. On the other hand, relatively high tyrosinase inhibitory activity was detected in *Inonotus mikadoi* (IMI), *Coriolus versicolor* (CVE), *Volvariella volvacea* (VVO), *Panellus serotinus* (PSE), *Auricularia auricula* (AAU), and *Fomitopsis* sp. (Fsp). Interestingly, the APO EECiM exhibited the highest DPPH radical scavenging rate (77.5 ± 4.3%) and reducing power (1.18 ± 0.041), while the highest inhibitory power of the TBA value and antityrosinase activity were detected in that of TAU (64.5 ± 4.1%) and IMI (46.0 ± 7.5%), respectively. Overall, our study suggested potential candidates for EECiMs that exhibited powerful antioxidant and tyrosinase inhibitory properties and might be used as natural antioxidant tyrosinase inhibitor.

## 1. Introduction

Antioxidants are able to inhibit the oxidation of biomolecules by reducing the reactive oxygen species (ROS) level to prevent the damage caused by free radicals in body cells. Free radicals destructively react with biomolecules, inducing diseases such as cancer [1], aging [2], and atherosclerosis [3]. Antioxidants may prevent lipid oxidation in cell membrane through inhibition of the attack of free radicals, resulting in increased ability to defend against cellular damage [4]. Thus, taking supplements containing antioxidants is an alternative way of reducing oxidative stress. Since natural antioxidants have an inherent safety advantage, there is increasing interest concerning the use of natural antioxidants to replace synthetic antioxidants. These antioxidants can be separated from many natural products such as herbs [5, 6], vegetables [7], and fruits [8]. Mushrooms are also potential sources of antioxidants [9–11], and there is an increasing focus of therapeutic evidence for medicinal mushrooms in their use as anticancer [12–15] and antiviral [16, 17] agents.

Tyrosinase is key enzyme of melanogenesis. The antityrosinase activity of mushrooms has also been studied for the developing of functional cosmetics, because the inhibition of tyrosinase can attenuate the synthesis of melanin to have a whitening effect [18]. Antioxidants such as ascorbic acid and its derivatives have been reported to have whitening effect, indicating that melanogenesis could be prevented by the reduction of ROS levels in melanocytes [19]. Tyrosinase is also responsible for the browning of fruits and vegetables [20]. Thus, there has been considerable interest in tyrosinase inhibitors in the food industry and the needs for natural tyrosinase inhibitors have gradually been increased because safety is strictly monitored in the food industry.

Many studies have suggested the antioxidant properties of different mushrooms [21, 22]. These studies have generally used mushroom extracts; little attention has been paid to demonstrating the antioxidant activities or antityrosinase activity of mycelium culture supernatants. Recently, secretome analysis has been carried out extensively because the secreted proteins of the mycelium were found to have roles in pathogenesis [23, 24]. Interestingly, one report focused on the antioxidant activity from mycelium-free broths of* Phellinus igniarius* [25], and Tsai et al. reported that the spent culture supernatant of* Lactobacillus rhamnosus* could be used as an attractive source for making cosmetics with antioxidant and antityrosinase activity [26]. These results suggest that mycelium culture media may include ingredients that have antioxidant and antityrosinase activity. Accordingly, we considered that investigating antioxidant and antityrosinase activity from a large number of mycelium culture extracts would be a prerequisite for developing efficient natural antioxidants and tyrosinase inhibitors.

There are more advantages in using the mycelium rather than the fruit body of the mushroom when making products, because the mycelium can easily be produced and is inexpensive compared to cultivating mushrooms. Moreover, using the mycelium could be a more controllable way to develop large-scale products than mushroom cultivation. Consequently, the objective of this study was to determine the potential mycelium culture extracts that have antioxidant and tyrosinase inhibitory properties. Antioxidant activities were determined by testing the scavenging abilities on DPPH, reducing power, and inhibitory power of lipid oxidation. The tyrosinase inhibitory activity of each mycelium culture extract was also examined.

## 2. Material and Methods

### 2.1. Materials

Ethanol was purchased from Merck (Darmstadt, Germany) and vitamin C, vitamin E, catechin, arbutin, butylated hydroxytoluene (BHT), and DPPH were purchased from Sigma-Aldrich Co. (St. Louis, MO, USA).

### 2.2. Mycelium Culture and Preparation of Extract

Mycelium strains were obtained from National Academy of Agricultural Science in Korea. Each strain was first inoculated to potato dextrose agar plate (PDA) (Difco, Detroit, MI, USA) and the growing 7 mm size of each mycelium was cut and transferred to 100 mL of potato dextrose broth (PDB, pH 5.0) (Difco, Detroit, MI, USA). After incubation for 5 days at 25°C, mycelium of each strain was homogenized and inoculated again to 500 mL of PDB. Culture media including mycelium were homogenized and extracted with 5 volumes of 40% ethanol for 72 hours at 25°C. Extract was then concentrated with rotary evaporator and filtered with 0.45 um membrane filter (Advatec MFS Inc., Japan). Concentrated extract was freeze-dried and used for experiments.

### 2.3. DPPH Scavenging Activity Assay

DPPH scavenging activity was determined according to the previously described [27]. 0.2 mL of various concentrations of each EECiM (10, 1, 0.1 mg/mL), vitamin C (0.1 mg/mL), or BHT (0.1 mg/mL) was added to DPPH solution (40 *μ*M, 0.8 mL), respectively, and incubated for 10 min at room temperature. Absorbance at 517 nm was measured by UV-Vis spectrophotometer (Shimadzu Co. Tokyo, Japan). DPPH scavenging activity (*I*%) was calculated as *I*% = [1 − (*A*_sample_ − *B*_control_)/*C*_control_] × 100, where *A*_sample_ is the absorbance of the sample reaction (containing all reagents and test compound), *B*_control_ is the absorbance of the test compound, and *C*_control_ is the absorbance of control reaction (containing all reagents without test compound). Vitamin C and BHT were used as positive controls.

### 2.4. Measuring the Reducing Power

Reducing power of EECiM was measured according to the method of Oyaizu [28]. 0.2 mL of various concentrations of each EECiM (10, 1, 0.1 mg/mL), vitamin C (0.1 mg/mL), vitamin E (0.1 mg/mL), or BHT (0.1 mg/mL) was mixed with 0.5 mL of phosphate buffer (0.5 M, pH 6.0) and 0.5 mL of 1% (w/v) potassium ferricyanide [K_3_Fe(CN_6_)], respectively. After incubating the mixture for 20 min at 50°C, 0.5 mL of trichloroacetic acid (TCA) (10% w/v) was added and centrifuged for 10 min at 1,000 g (Sigma, Mannheim, Germany). Supernatant (0.5 mL) was mixed with distilled water (0.5 mL) and FeCl_3_ (0.1% w/v, 0.5 mL). Absorbance at 700 nm (A700) was measured by using UV-Vis spectrophotometer (Shimadzu. Tokyo, Japan). Vitamin C, Vitamin E, and BHT were used as positive controls.

### 2.5. Determination of TBA Value

TBA value was determined according to the method of Lin and Chang [29]. 10 mg/mL of each EECiM (0.4 mL) and BHT (0.1 mg/mL) was mixed with 1 mL of linoleic acid emulsion [linoleic acid solution (60% w/v, 0.1 mL), tween 20 (0.2 mL), distilled water (19.7 mL)], 0.5 mL of 20 mM PBS, and 0.2 mL of FeSO_4_ (0.01% w/v). Mixture was then added with 0.2 mL of FeSO_4_ (0.01% w/v) and 0.2 mL of H_2_O_2_ (0.56 mM) and incubated for 6 hours at 37°C. After incubation, 0.2 mL of butylated hydroxytoluene (BHT, 0.4% w/v), 0.2 mL of trichloroacetic acid (4% w/v), and 2 mL of 2-thiobarbituric acid (TBA, 0.8% w/v) were added and incubated for 30 min at 100°C. 2 mL of *n*-Butanol was added to mixture and then centrifuged at 200 g (Sigma, Mannheim, Germany). Absorbance at 532 nm was measured by using UV-Vis spectrophotometer (Shimadzu, Tokyo, Japan). TBA inhibitory rate (*I*%) was calculated: *I*% = [1 − (*A*_sample_ − *A*_control_)/*B*_control_] × 100, where *A*_sample_ is the absorbance of the sample reaction (containing all reagents and test compound), *A*_control_ is the absorbance of the test compound, and *B*_control_ is the absorbance of control reaction (containing all reagents without test compound).

### 2.6. Tyrosinase Inhibitory Activity

Tyrosinase inhibitory activity of each EECiM was determined as described previously [30]. Briefly, mushroom tyrosinase (20 *μ*L, 1700 unit/mL) was mixed with 220 *μ*L of phosphate buffer (0.1 M, pH 6.5) and 40 *μ*L of 1.5 mM tyrosine and 20 *μ*L of different concentrated EECiM (10, 1, and 0.1 mg/mL). The mixture was then incubated for 15 min at 37°C. Following incubation, absorbance of the mixture was determined at 490 nm by using a UV-Vis spectrophotometer (Shimadzu, Tokyo, Japan). Arbutin (10, 1, and 0.1 mg/mL) and vitamin C (10 mg/mL) were used as positive control in this study. Percent inhibition of tyrosinase activity was determined according to the formula: inhibition (%) = 100 − (*W*_sample_/*W*_blank_) × 100, where *W* is the absorbance at 490 nm. *W*_blank_ is the absorbance of control reaction (containing all reagents without test compound).

## 3. Results

### 3.1. Preparation of EECiMs

**Figure 1:**
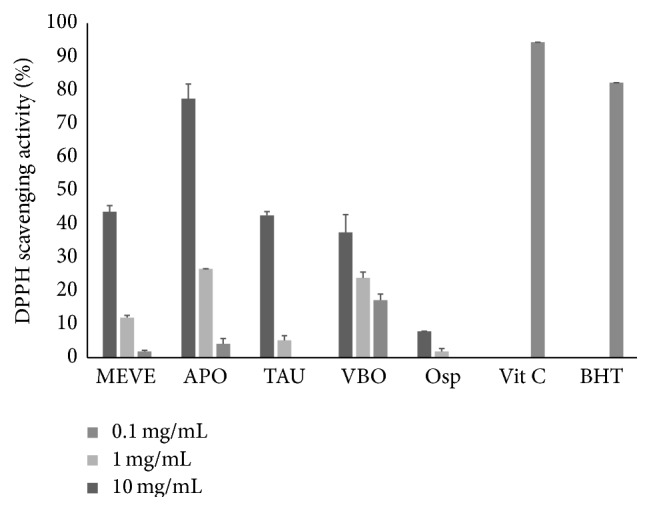
DPPH radical scavenging activities of five EECiM. Each EECiM at various concentrations (0.1, 1, 10 mg/mL), vitamin C (0.1 mg/mL), and BHT (0.1 mg/mL) was interacted with DPPH. Values represented as means ± SD (*n* = 3). MEVE, APO, TAU, VBO, and Osp represent the EECiM of* Morchella esculenta *var.* esculenta, Auricularia polytricha, Tremella aurantia, Volvariella bombycina, *and* Oudemansiella *sp., respectively.

**Figure 2:**
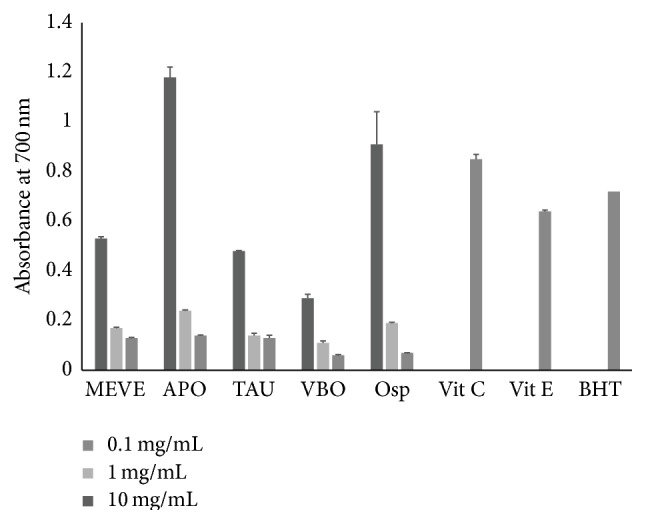
Reducing power of five EECiMs. Different concentrations of five EECiMs (0.1, 1, and 10 mg/mL), vitamin C (0.1 mg/mL), vitamin E (0.1 mg/mL), BHT (0.1 mg/mL) were used for investigating the reducing power. Results are represented as absorbance at 700 nm (A700) and values are expressed as means ± SD (*n* = 3). MEVE, APO, TAU, VBO, and Osp represent the EECiM of* Morchella esculenta *var.* esculenta, Auricularia polytricha, Tremella aurantia, Volvariella bombycina, *and* Oudemansiella *sp., respectively.

**Figure 3:**
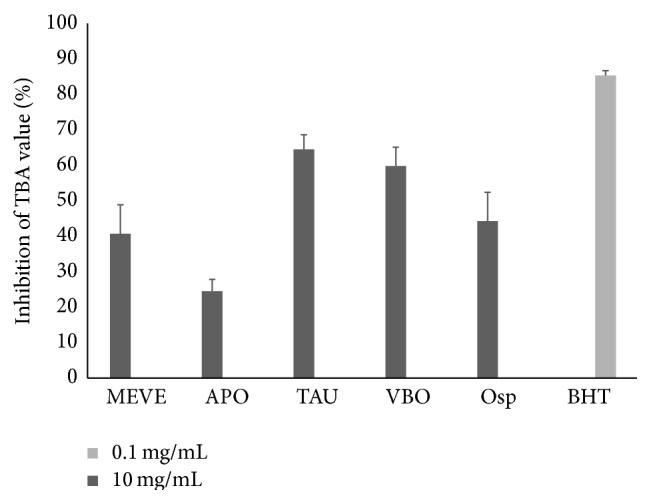
Inhibitory activity of TBA values of five EECiMs. 10 mg/mL of each EECiM and 0.1 mg/mL of BHT were used for determining the inhibitory activity of TBA value. Results are represented as inhibitory rate (%) of TBA value and expressed as means ± SD (*n* = 3). MEVE, APO, TAU, VBO, and Osp represent the EECiM of* Morchella esculenta *var.* esculenta, Auricularia polytricha, Tremella aurantia, Volvariella bombycina,* and* Oudemansiella *sp., respectively.

**Figure 4:**
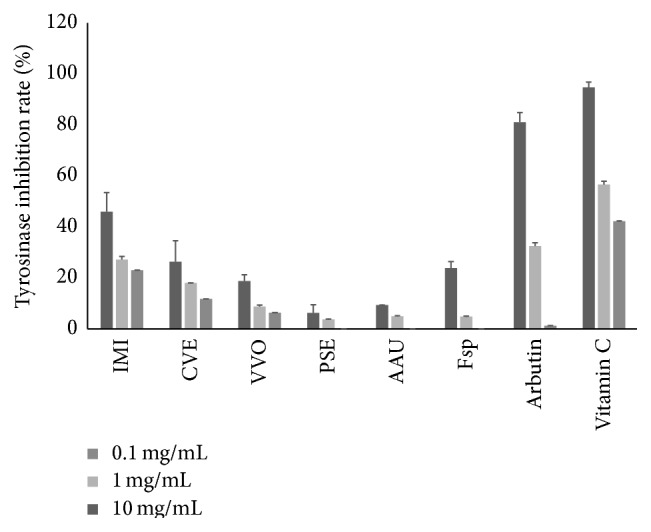
Tyrosinase inhibitory activities of EECiMs. Various concentrations (0.1, 1, and 10 mg/mL) of EECiM and arbutin were used for determining the tyrosinase inhibitory activity. Arbutin and vitamin C were used as positive control for measuring the tyrosinase inhibitory activity. Values represented as means ± SD (*n* = 3). IMI, CVE, VVO, PSE, AAU, and Fsp represent the EECiM of* Inonotus mikadoi*,* Coriolus versicolor*,* Volvariella volvacea*,* Panellus serotinus*,* Auricularia auricular,* and* Fomitopsis *sp., respectively.

Fifty-five EECiMs were prepared according to the method described in Section 2. Each EECiM was used for measuring the antioxidant activities and tyrosinase inhibitory properties. As shown in Figures 1–4, several EECiMs showed strong antioxidant activities and tyrosinase inhibitory property indicated that mycelium including culture media could be a useful source for preparing the natural antioxidant.

### 3.2. Several EECiMs Showed Strong DPPH Scavenging Activity

The DPPH assay is a well-known method that is used to determine antioxidant properties, because DPPH can donate hydrogen when DPPH reacts with an antioxidant, resulting in a change in color. Thus, the antioxidant activities of each EECiM were first screened by measuring the DPPH scavenging ability. Most EECiM showed low or negligible DPPH scavenging activity (data not shown) but EECiM containing MEVE, APO, TAU, VBO, and Osp showed relatively high DPPH scavenging activity (Figure 1 and Table 1). Although those DPPH scavenging activities were lower than that of vitamin C (94.3 ± 0.0% at a concentration of 0.1 mg/mL) and BHT (82.2 ± 0.0% at a concentration of 0.1 mg/mL), the EECiM of APO showed the highest DPPH scavenging activity (77.4 ± 4.4%) at a concentration of 10 mg/mL. Other EECiMs such as MEVE (43.7 ± 1.9%), TAU (42.6 ± 1.1%), and VBO (37.5 ± 5.4%) also showed DPPH scavenging activities at a concentration of 10 mg/mL. These data suggest that the five EECiMs that were found in this study have good potential as antioxidants.

### 3.3. APO EECiM Showed Highest Reducing Power

Since the antioxidant action was performed by reductones, which is associated with reducing power through the breakage of the free radical chain, we next investigated the reducing power of five EECiMs, which found at DPPH scavenging test, at various concentrations for each EECiM (0.1, 1, and 10 mg/mL), vitamin C (0.1 mg/mL), vitamin E (0.1 mg/mL), and BHT (0.1 mg/mL). As shown in Figure 2, all five EECiMs apparently exhibited reducing power that increased in a dose-dependent manner. The reducing powers (A700) of the APO EECiM, which showed the highest reducing power from among the five EECiMs, were 0.14 ± 0.002, 0.24 ± 0.003, and 1.18 ± 0.041 at concentrations of 0.1, 1, and 10 mg/mL, respectively. Meanwhile, the reducing powers (A700) of vitamin C, vitamin E, and BHT at a concentration of 0.1 mg/mL were 0.85 ± 0.019, 0.64 ± 0.005, and 0.72 ± 0.003, respectively.

### 3.4. EECiMs Showed Inhibitory Activity of Lipid Oxidation

Since thiobarbituric acid (TBA) can form a complex with malondialdehyde (MDA), which is a major product of oxidized lipid materials, the TBA–MDA complex has been used to determine the degree of lipid oxidation in biological tissue or food [31]. To evaluate the inhibitory effect of EECiMs during lipid oxidation, the TBA value was determined by adding each EECiM, which found by DPPH scavenging test, to the linoleic acid peroxidation reaction. As shown in Figure 3, five EECiMs showed a strong inhibitory effect on linoleic acid peroxidation ranging from 24.5% to 64.5%. Interestingly, the APO EECiM, which showed the highest DPPH scavenging activity and reducing power (Figures 1 and 2), was shown to have the lowest inhibitory activity during linoleic acid peroxidation (24.5 ± 3.3%) from among the five EECiMs (Figure 3). These data indicate that the five EECiMs, which showed in Figure 3, have inhibitory potential during lipid oxidation and could be used to develop antilipid oxidants.

### 3.5. Antityrosinase Activity Was Detected in EECiMs

Since the tyrosinase has an important role in melanin synthesis, the materials which have an inhibitory activity of tyrosinase are considered as good candidate for getting whitening effect. So, we next investigated the tyrosinase inhibitory potentials of 55 EECiMs at various concentrations for each EECiM (0.1, 1, and 10 mg/mL), arbutin (0.1, 1, and 10 mg/mL), and vitamin C (0.1, 1, and 10 mg/mL). Interestingly, EECiMs which showed high antioxidant properties (Figures 1, 2, and 3) exhibited very low tyrosinase inhibitory activity (<3% at 10 mg/mL concentration). Relatively high tyrosinase inhibitory activity was detected from EECiMs containing IMI (46.0 ± 7.5%), CVE (26.3 ± 8.3%), VVO (18.8 ± 2.5%), PSE (6.3 ± 3.2%), AAU (9.4 ± 0.0%), and Fsp (23.9 ± 2.5%) at the concentration of 10 mg/mL (Figure 4).

## 4. Discussion

This study examined the antioxidant properties and antityrosinase activity of 55 different mycelium culture extracts. Among them, five EECiMs of MEVE, APO, TAU, VBO, and Osp were selected due to their reliable antioxidant properties, and EECiMs of IMI, CVE, VVO, PSE, AAU, and Fsp were determined to have relatively high tyrosinase inhibitory activity. Although our study proposed a preliminarily investigation of antioxidant and antityrosinase activity, to our knowledge, this is the first report demonstrating those properties with each mycelium culture extract. Since the use of mycelium culture extracts is advantageous in large-scale production, we considered that a preliminary investigation of medicinal properties such as antioxidant and antityrosinase activity was required for a large number of different strains. Although the actual components which represent these properties could not be identified in this study, if we consider that most papers have focused on analyzing one or two strains to elucidate their antioxidant property, our study contributes significant information about EECiM candidates that have strong antioxidant or antityrosinase activity.

Until now, research on the medicinal properties of APO—which showed the highest DPPH scavenger activity in this study (Figure 1)—seems to have mostly focused on its anticancer effects [32]. Although the antihypercholesterolemic effect of APO was recently reported [33], little attention has been paid to the antioxidant property of APO culture extract. In addition, the EECiM of APO was revealed to have strong reducing power (Figure 2), as well as TBA inhibitory activity (Figure 3). Because mycelium culture in liquid medium has an advantage in quantitative biomass production, our study strongly suggests that EECiM of APO has an advantage in developing natural antioxidants.

In the case of TAU, only a few studies have demonstrated its effect on glucose metabolism, including its antidiabetic effect [33]. TAU polysaccharide seems to have critical roles in regulating the enzyme activity related with glucose metabolisms [34]. In our study, EECiM of TAU showed antioxidant activity. Interestingly, EECiM of TAU revealed the highest inhibitory effect on lipid oxidation (Figure 3). Functional analysis of TAU polysaccharide on lipid oxidation will give us a deeper understanding of the regulatory role of EECiM of TAU in lipid oxidation.

Tyrosinase is a key enzyme of melanogenesis and is also called polyphenol oxidase. Since tyrosinase inhibitors can be used as effective skin whitening agents, many natural sources such as grape seed and raspberry have been studied to investigate their antityrosinase activity [35]. Although the actual components that have antityrosinase activity were not identified in this study, we found several attractive EECiM candidates which could be used as natural tyrosinase inhibitors. Among them, EECiM containing IMI showed the highest tyrosinase inhibitory property among the 55 different EECiMs, and about 45% of tyrosinase activity was inhibited at a concentration of 10 mg/mL. Although one report detected lipoxygenase inhibitory activity from IMI [36], IMI is a rarely characterized species. In our study, EECiMs of IMI and CVE showed high tyrosinase inhibitory activity (Figure 4). Interestingly, EECiM of IMI showed the highest astringency effect among 55 different EECiMs, and CVE also showed a high astringency effect (data not shown). Taken together, these data strongly suggested that EECiMs that are selected to have tyrosinase inhibitory activity could be developed as functional additives for cosmetics.

Consequently, our study proposed several candidates for natural antioxidants and tyrosinase inhibitors. Although we found that vitamin C has strong antioxidant and antityrosinase activities (Figures 1, 2, and 4), we could not find EECiMs which have strong both antioxidant and antityrosinase activities. This fact indicated that each EECiM possesses different components to show antioxidant and antityrosinase properties. The* in vitro* antioxidant activity of mushroom extracts seems to be closely correlated with its polysaccharide contents [37] or phenolic components [38]. Several studies have suggested natural tyrosinase inhibitors such as flavonoids [39] and phloroglucinol derivatives [40]. Analysis of active components of the EECiMs selected in this study will give us a deeper understanding of the mechanisms of antioxidant and tyrosinase inhibition of EECiMs. Fractionation to isolate the active components of EECiMs and further identification are in progress.

## 5. Conclusion

In this study, we found the several mycelia which have DPPH radical scavenging activity, reducing power, inhibitory activity of TBA value, and tyrosinase inhibition. In addition, we included the culture media of each mycelium in making extracts and showed strong antioxidant activities and a tyrosinase inhibitory property, which indicated that EECiM is a useful source for making antioxidants. Thus, the EECiMs that were determined to have antioxidant activities and a tyrosinase inhibitory property in our study can be used as additives in natural antioxidants and functional cosmetics.

## Figures and Tables

**Table 1:** Mycelium strains which are used in this study.

	Genus	Species
1	*Auricularia *	sp.
2		*auricula-judae *
3		*polytricha *
4		*auricula *

5	*Cerrena *	*unicolor *

6	*Clitocybe *	*maxima *
7		*aurantiaca *

8	*Collybia *	*abundans *
9		*peronata *
10		*confluens *
11		*erythropus *
12		aquosa

13	*Mycena *	pelianthina

14	*Coprinus *	*cinereus *
15		*comatus *

16	*Coriolus *	*brevis *
17		*hirsutus *
18		*versicolor *

19	*Dacrymyces *	*palmatus *

20	*Fomes *	sp.
21		*fomentarius *

22	*Fomitella *	*fraxinea *

23	*Fomitopsis *	*roseus *
24		pubertasis
25		sp.
26		*pinicola *

27	*Hericium *	*erinaceus *

28	*Hohenbuehelia *	*petaloides *
29		sp.
30		*myxotricha *

31	*Inonotus *	*xeranticus *
32		*hispidus *
33		*obliqua *
34		*mikadoi *

35	*Macrolepiota *	*alborubescens *

36	*Morchella *	*esculenta *var*.umbrina *
37		*esculenta *var*.esculenta *
38		*esculenta *
39		*elata *

40	*Oudemansiella *	*mucida *
41		*radicata *
42		sp.
43		*pudens *
44		brunneomarginata

45	*Panellus *	*serotinus *
46		*stipticus *
47		sp.

48	*Phyllotopsis *	*nidulans *

49	*Rhodotus *	*palmatus *

50	*Wolfiporia *	*cocos *

51	*Schizophyllum *	*commune *

52	*Tremella *	sp.
53		*aurantia *

54	*Volvariella *	*bombycina *
55		*volvacea *
